# A novel GDF1 frameshift mutation in a young adult with right coronary artery hypoplasia and myocardial bridging: a case report

**DOI:** 10.3389/fcvm.2026.1797320

**Published:** 2026-05-05

**Authors:** Zetong Xiang, Xincheng Wu, Huishuang Chen, Lijuan Kan, Qiuxia Ding

**Affiliations:** 1 Department of Urology, The Third Affiliated Hospital of Shenzhen University (Luohu Hospital Group), Shenzhen University, Shenzhen, China; 2 Luohu Clinical Institute, Shantou University Medical College, Shantou, Guangdong, China; 3 Medical Laboratory, Shenzhen Luohu People's Hospital, Shenzhen, Guangdong, China

**Keywords:** GDF1, hypoplastic coronary artery disease, myocardial bridging, frameshift mutation, congenital coronary anomaly, case report

## Abstract

**Background:**

Hypoplastic Coronary Artery Disease (HCAD) is a rare congenital malformation. While linked to genes like NOTCH1, its genetic spectrum is incomplete. No prior association with GDF1 exists.

**Case presentation:**

A 24-year-old male with exertional chest pain underwent coronary computed tomography angiography (CCTA), which revealed right coronary artery (RCA) hypoplasia (diameter <1.5 mm) and a superficial myocardial bridge (SMB) of the left ramus artery. Whole-exome sequencing identified a novel heterozygous frameshift mutation in GDF1 (NM_001492.4:c.84_91del; p.Val31ArgfsTer15), validated by Sanger sequencing. According to ACMG guidelines, the variant was classified as likely pathogenic (PVS1 + PM2).

**Conclusion:**

This is the first report suggesting a potential association between a GDF1 loss-of-function mutation and HCAD and SMB, expanding the phenotypic spectrum of GDF1-related disorders and providing insights into the genetic etiology of congenital coronary anomalies.

## Introduction

Hypoplastic Coronary Artery Disease (HCAD), first reported in 1970 (1), is a rare congenital anomaly characterized by underdevelopment of one or more major coronary branches, manifesting as significantly reduced lumen diameter or length (1, 2). It is confirmed via coronary angiography (CAG), CCTA, which reveal diffusely thinned coronary arteries (with normal left main diameter ∼4–5 mm, left anterior descending/circumflex ∼3–4 mm) (3). Villa et al. defined it as congenital reduction in coronary trunk/branch diameter, accompanied by fewer branches or smaller perfusion areas (4).

Currently, there is no unified diagnostic consensus or guidelines for coronary artery developmental abnormalities. However, previous studies have proposed that a coronary lumen diameter <1.5 mm serves as a key indicator of developmental anomalies (5). Specifically, HCAD is diagnosed via CTA when findings include lumen diameter narrowing or shortened course, with a lumen diameter <1.5 mm and the absence of compensatory branches in the vicinity (6, 7). Clinically, HCAD manifests variably depending on severity: mild cases may be asymptomatic, while severe cases can present with syncope, dyspnea, chest discomfort, dizziness, or even progress to ischemic heart disease, heart failure, or sudden cardiac death (8).

While mutations in genes such as NOTCH1, HAND1 and NKX2-5 have been implicated, the genetic etiology of HCAD remains largely unknown (9–11). Growth differentiation factor 1 (GDF1), a member of the TGF-β superfamily, plays a critical role in embryonic cardiovascular patterning. However, its association with isolated coronary artery microstructural anomalies has not been established. Herein, we present the first case suggesting a potential link between a novel, likely pathogenic GDF1 frameshift mutation and the clinical phenotype of RCA hypoplasia and concomitant SMB.

## Case description

A 24-year-old male of Chinese Han ethnicity presented to the Cardiology Department of the Third Affiliated Hospital of Shenzhen University (Luohu Hospital Group) with recurrent exertional chest tightness, dyspnea, and angina. Symptoms were typically triggered by emotional stress or physical exertion and consistently relieved by rest within 5–10 min. The patient had no significant past medical history and was not taking any regular medications.

On admission, vital signs were stable: blood pressure 118/76 mmHg, heart rate 72 beats/min, respiratory rate 16 breaths/min, oxygen saturation 98% on room air. Cardiovascular examination revealed a regular heart rhythm without murmurs, rubs, or gallops. No peripheral edema, cyanosis, or jugular venous distension was observed. Initial resting ECG findings are detailed in the Electrocardiographic and Biomarker Findings subsection below.

The patient reported no known family history of congenital heart disease, sudden cardiac death, or inherited cardiovascular conditions among first-degree relatives. His parents and younger sibling are reportedly healthy, though no genetic testing has been performed on family members.

The patient initially expressed aversion to invasive coronary angiography but agreed to non-invasive diagnostic evaluations, including serial electrocardiography and cardiac biomarker testing. Diagnostic workup included CCTA, whole-exome sequencing (WES), and Sanger sequencing, which identified a heterozygous GDF1 frameshift mutation (NM_001492.4:c.84_91delCGCCCCCG, p.Val31ArgfsTer15).

### Electrocardiographic and biomarker findings

During hospitalization, the patient underwent serial electrocardiography (ECG) and high-sensitivity cardiac troponin I (hs-cTnI) monitoring. On admission (July 10, 2021), the ECG revealed pathological Q waves in the inferior leads (II, III, aVF) without ST-segment elevation. The hs-cTnI level was 0.02 ng/mL (within normal limits). On July 11, the inferior Q waves persisted, and the ECG showed flattened T waves in leads V4–V6 with no ST-segment shifts; hs-cTnI had risen to 0.38 ng/mL. On July 13, the inferior pathological Q waves remained present, accompanied by mild T-wave inversions in leads II, III, and aVF, while hs-cTnI reached a peak of 1.26 ng/mL. By discharge (July 17), the inferior Q waves were unchanged, the T-wave inversions had improved, and hs-cTnI had returned to normal levels. The combination of persistent inferior pathological Q waves and a dynamic rise-and-fall pattern of hs-cTnI is suggestive of acute myocardial injury potentially related to the vascular territory of the hypoplastic right coronary artery (Table 1).

**Table 1:** Electrocardiographic and biomarker findings.

Time point	ECG findings	hs-cTnI (ng/mL)	CK-MB (ng/mL)	Myoglobin (ng/mL)
2021-07-10 (admission)	Pathological Q waves in inferior leads (II, III, aVF); no ST-segment elevation	0.02	2.1	45
2021-07-11	Inferior Q waves persist; flattened T waves in V4–V6	0.38	3.5	68
2021-07-13	Inferior Q waves present; mild T-wave inversions in II, III, aVF	1.26	2.8	52
2021-07-17 (discharge)	Inferior Q waves unchanged; T-wave inversions improved	0.03	2.2	43
Reference range	–	0–0.04	0–4.3	<70

The reference ranges provided above are based on the clinical laboratory standards of our hospital. For other institutions, these values may vary.

### Diagnostic assessment (Imaging)

**Figure 1:**
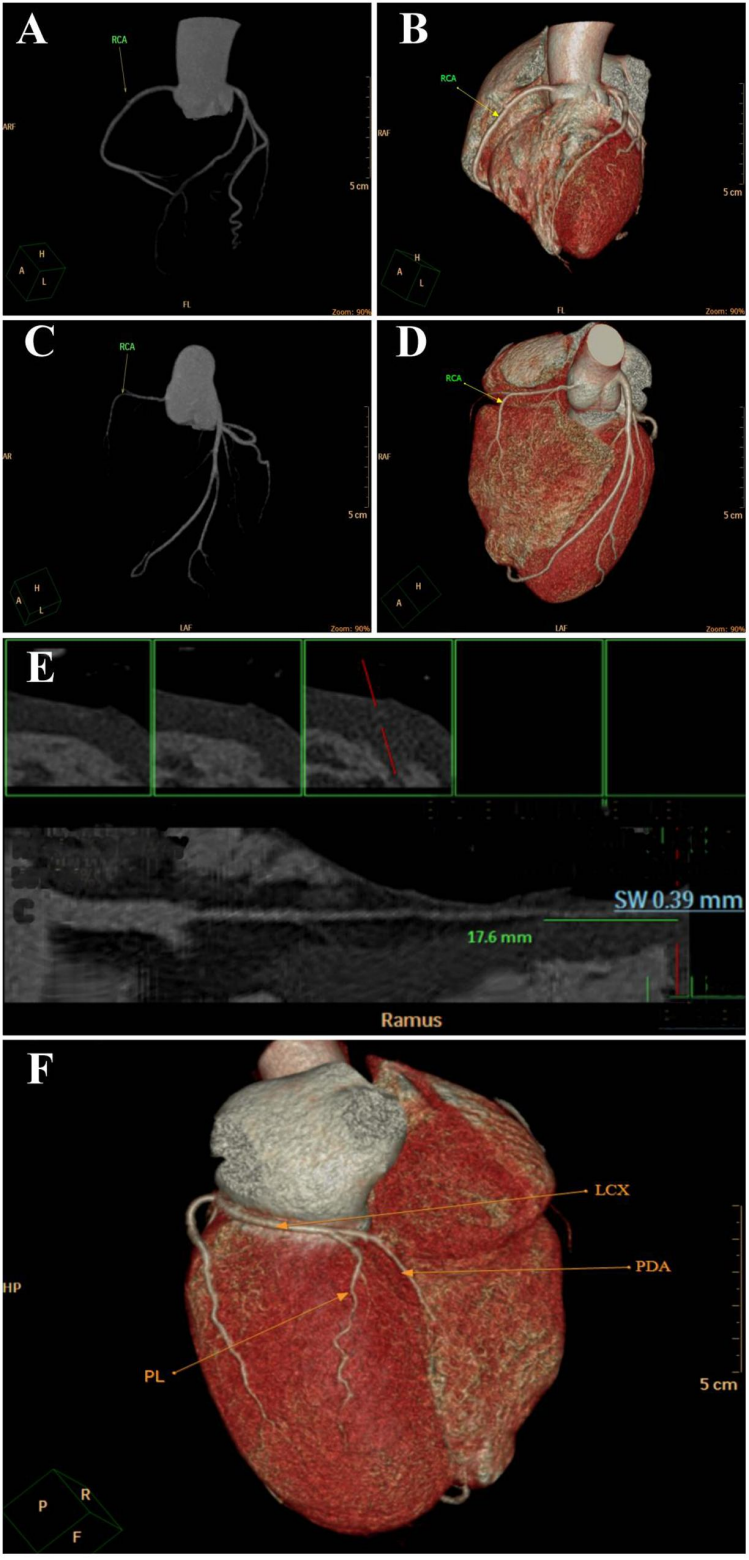
CCTA of the patient in this instance **(C,D)** was compared with that of a normal individual **(A,B)**, revealing that the RCA in both C and D is significantly shorter than that in A and B. Furthermore, within the patient's own CTAA, the RCA is noticeably shorter than the LCA, exhibiting a marked shortness with a distinct narrowing in the distal segment, indicating evident hypoplasia.; **(E)** SMB was identified at the site of the LCA. The associated subintimal wall thickness was measured at 0.39 mm, and the length of the affected segment was 17.6 mm; **(F)** CTAA of the patient shows the PL and PDA were identified to originate from the LCX: G: The diameter of RCA is less than 1.5 mm throughout the whole process.

**Figure 2:**
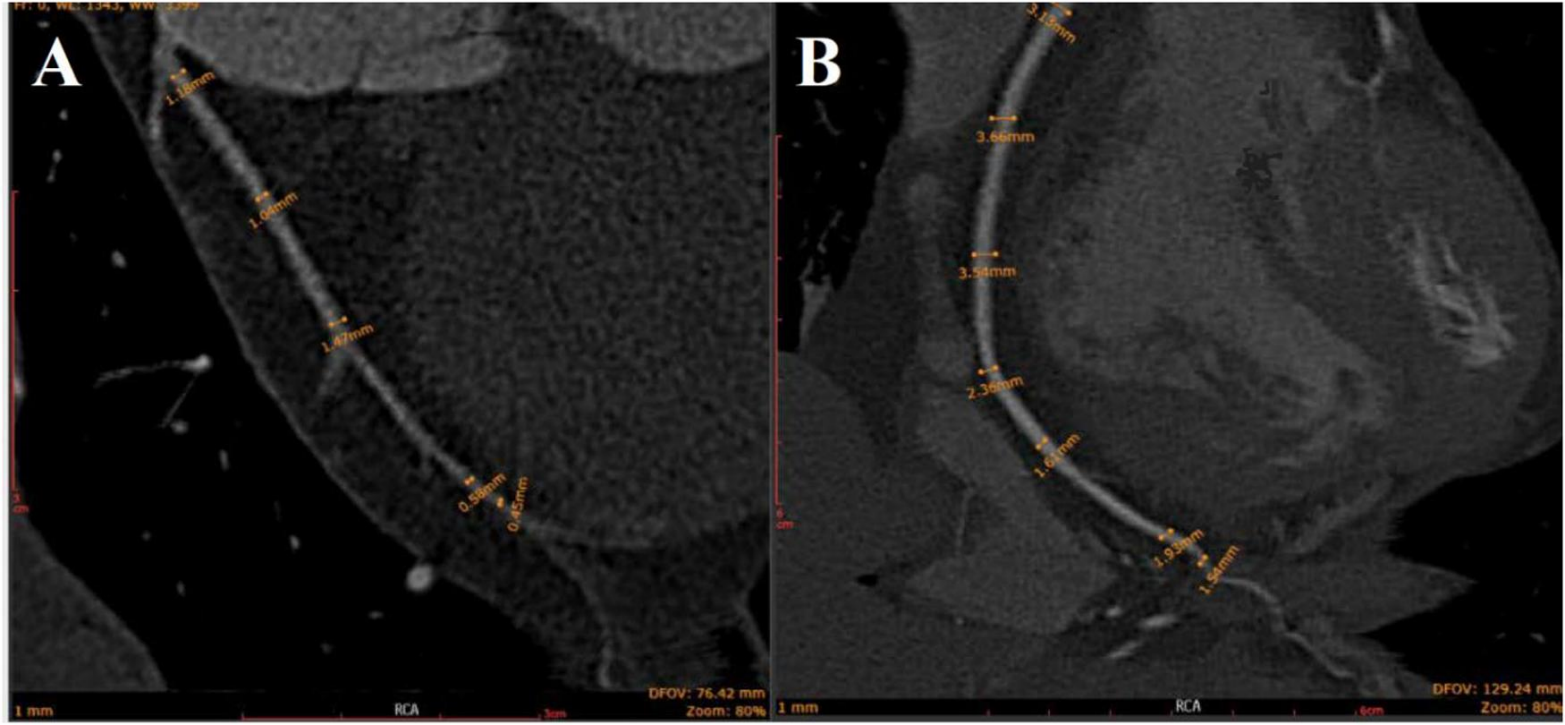
As shown in **(A)** (patient) and **(B)** (normal control), the diameter of each marked lumen segment in the patient's RCA is <1.50 mm, while that at the corresponding site in the normal RCA is >1.50 mm.

CCTA demonstrated a diffusely hypoplastic RCA with a maximum diameter of <1.5 mm along its course (Figures 1C,D, 2A), compared to normal controls (Figures 1A,B, 2B). A superficial myocardial bridge involving the left ramus artery was also observed, with a length of 17.6 mm and a depth of 0.39 mm (Figure 1E).

### Diagnostic assessment (Genetic)

**Figure 3:**
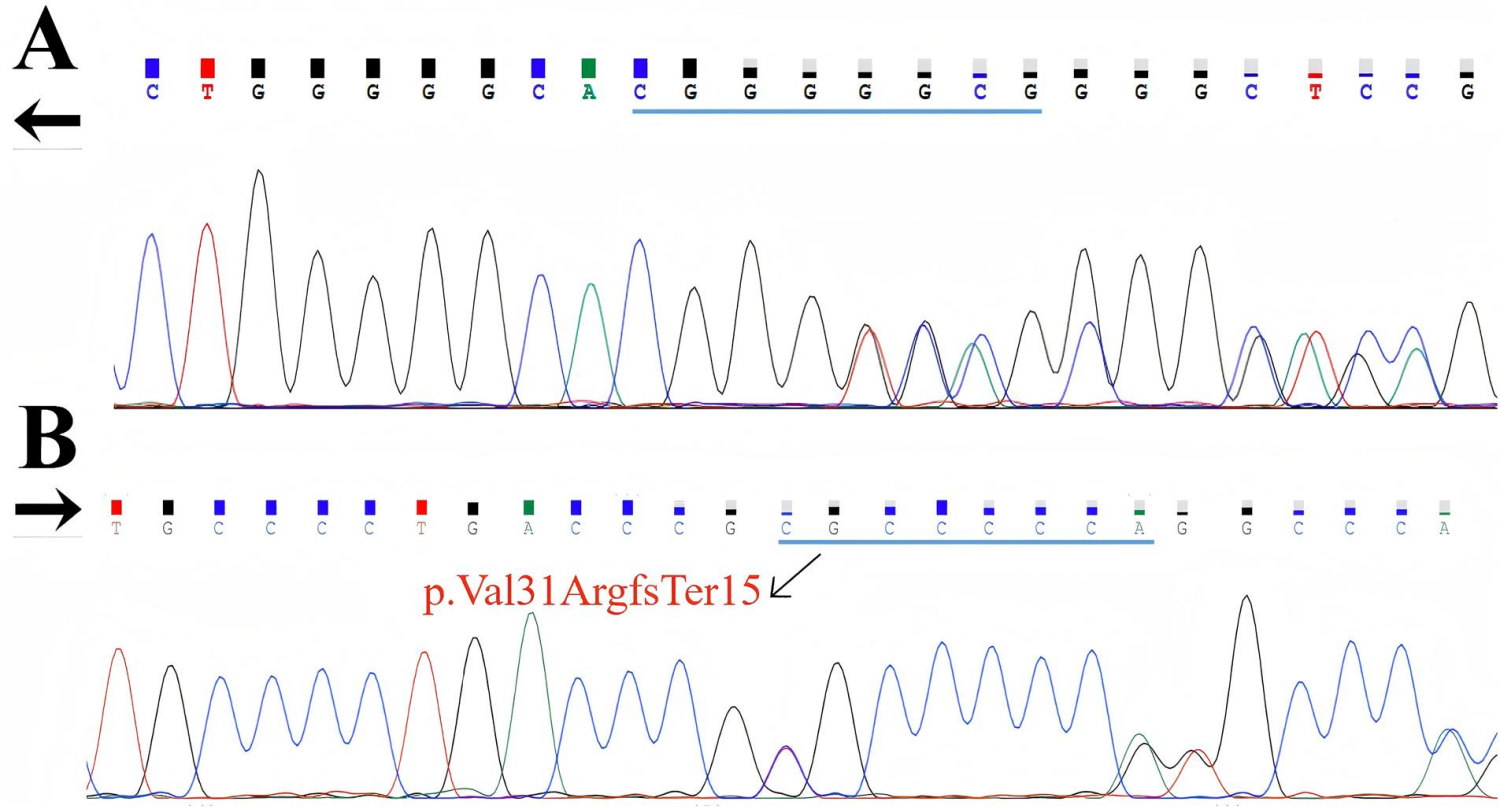
Illustrate a mutation site analysis in a gene, highlighting a frameshift mutation due to a single nucleotide insertion, which introduces a premature termination codon at protein position 31, resulting in a truncated protein product designated as p.Val31ArgfsTer15; **(A)** reverse sequencing, **(B)** forward sequencing.

WES revealed a heterozygous frameshift deletion in GDF1 (c.84_91del; p.Val31ArgfsTer15) (Figure 3). This variant was absent in population databases (gnomAD v4.1.0) and has not been reported in ClinVar or the literature. According to the ACMG/AMP guidelines, this variant meets criteria for classification as likely pathogenic:

1. PVS1 (null variant in a gene where loss-of-function is a known mechanism of disease): The frameshift deletion introduces a premature termination codon at amino acid position 31 (p.Val31ArgfsTer15), predicted to result in nonsense-mediated mRNA decay or production of a severely truncated, non-functional GDF1 protein. Given that GDF1 haploinsufficiency is an established mechanism in congenital heart disease, this variant is highly likely to result in loss of function.
2. PM2 (absent from population databases): The variant is absent from gnomAD (GRCh38 v4.1.0), which contains over 1.6 million alleles, indicating it is extremely rare in the general population.

Functional prediction tools further support pathogenicity: the CADD score is 25.3 (exceeding the recommended threshold of 20), and the MSC_95 (Mutation Significance Cutoff at the 95% confidence interval) for GDF1 is 19.6, with the variant score significantly exceeding this gene-specific cutoff. Collectively, these data support a loss-of-function mechanism underlying the likely pathogenicity of this novel variant.

### Follow-up and outcomes

The patient was advised on lifestyle modifications, including avoidance of strenuous physical activity and emotional stress, along with annual comprehensive cardiovascular evaluation. At 6-month follow-up, the patient reported significant reduction in symptom frequency, with only occasional mild chest tightness during periods of high stress. He reported good adherence to lifestyle recommendations. No further diagnostic testing was performed during this period as the patient remained clinically stable and continued to decline invasive procedures. A repeat ECG showed no interval changes. The patient continues to be followed annually, with no adverse events reported to date.

## Discussion

In previous reports of GDF1 mutations, pathogenic variants are predominantly clustered in exon 8 (e.g., NM_001492.6:c.485G>A [p.Gly162Asp], NM_001492.6:c.681C>A [p.Cys227Ter]). which encodes the C-terminal domain of the GDF1 protein, a critical region for its regulatory activity. In contrast, the mutation identified in this study, c.84_91delCGCCCCCG (p.Val31ArgfsTer15), is located in the 5′-proximal region of exon 7. The deletion results in the loss of a critical sequence within the coding region of the gene, leading to a frameshift mutation at amino acid position 31 (denoted as p.Val31ArgfsTer15), resulting in premature termination codon and protein truncation. Based on the comprehensive scores from MSC, CADD, and pLI, this variant is predicted to result in a high likelihood of functional loss of the GDF1 protein. No identical mutations or cases of HCAD associated with GDF1 mutations have been reported in mainstream mutation databases (gnomAD, ClinVar) or literature (PubMed) and according to ACMG guidelines, this mutation meets the PVS1 + PM2 standards, indicating the high novelty and rarity of this mutation.

Comprehensive analysis of clinical reports in patients with GDF1 mutations revealed no documented cases linking these mutations to coronary artery developmental anomalies. Notably, while prior studies predominantly describe structural cardiac defects (e.g., tetralogy of Fallot [TOF], atrial septal defect [ASD]), two cases of major vascular anomalies—transposition of the great arteries (TGA) and total anomalous pulmonary venous drainage (TAPVD)—have been reported in case report 1,5,6 (Tables 2) (12–18). These findings align with GDF1's established role in vascular development, particularly its dual mechanisms: (1) directing the migration of vascular progenitor cells and (2) driving their differentiation into mesenchymal cells that subsequently form vascular structures. By integrating molecular biology studies of GDF1 in early vascular development with related clinical reports, we propose that hypoplasia of the RCA in this case may be associated with the identified GDF1 mutation. This represents the first documented case elucidating GDF1's role in coronary microstructural abnormalities.

**Table 2:** Summary of the exon section and vicinity in some previous case reports.

Number	Protein Changes	Exon	Basic Information	Survival Status	Cardiac Issue	*GT	*MT
1	p.Met364Thr	exon8	one fetus	miscarriage	IV*, DORV*, PS*, RAA*	Hom*	mis*
			one 9-month-old male infant	survival	DORV, TA*, PA*, APVD*	Het*	mis
2	*p.Cys227Ter/c.909insC	exon8	one 1-year-old female infant	death	TAPVD*, UVAVC*, MGA*, PS	Hom	un*
			one 2-year-old male infant				
			one male newborn				
			one male newborn				
			one 1-year-old male infant				
3	p.Cys227Ter	exon8	seven people with unknown information	survival	TGA*	un	non*
	p.Gly162Asp				TOF*		mis
	p.Arg68His				AV Canal with Cleft MV, Left superior vena cava to coronary sinus, coarctation of the aorta		
	p.Cys267Tyr				DORV,LPA-S*		
	p.Ala318Thr				TGA		
	P312T(P59T)			un	TOF		
	p.Ser309Pro				TOF,VSD*, AoRD*, Bi-PS*		
4	*p.Ala118Val	exon8	nineteen infants	uncertain*	CTDs*	Het	mis
			two infants		CTDs		
			fifteen infants		AVSD*		
			one infant		APVR*		
			eight infants		LVOTO*		
			three infants		RVOTO*		
			one infant		Heterotaxy	Hom	
			one infant		LVOTO		
5	p.Met364Thr	exon8	ten Ashkenazi Jewish people	survival	L-DORV*	Hom	mis
					CCTGA*		
					L-TGA*		
					TOF		
					D-TGA*		
					DORV, D-TGA		
					TOF		
					CCTGA		
					D-TGA		
					D-TGA		
6	p.Trp203Ter	exon8	one person with unknown information	survival	TGA	Het	non
			one Arab Muslim one-month-old male infant	death	SV with SAV	Hom	
			one Arab Muslim one-month-old male infant		TGA, HLHS		
			one Arab Muslim nine-month-old infant		TGA		
7	p.Ala266Val	exon8	one Chinese patient	survival	RAI, RAA type3	Het	mis

GT, Genotype; MT, mutation, un, unknown; IV, Inversion Ventricular; DORV, Double outlet right ventricle; PS, Pulmonary stenosis; RAA, Right aortic arch; Hom, Homozygous; mis, missense, non, nonsense; Het, Heterozygous; TA, Tricuspid Atresia; PA, Pulmonary Atresia; APVD, Anomalous pulmonary venous drainage, TAPVD, Total anomalous pulmonary venous drainage; UVAVC, Univentricular atrioventricular connection; MGA, Malposition of the great arteries; TOF, Tetralogy of Fallot; TGA, Transposition of the great arteries; LPA-S, Left pulmonary artery stenosis; VSD, Ventricular septal defect; AoRD, Aortic root dilation; Bi-PS, Bilateral pulmonary stenosis; CTDs, conotruncal defects; AVSD, Atrioventricular Septal Defect; APVR, Anomalous Pulmonary Venous Return; LVOTO, Left ventricular outflow tract obstruction; RVOTO, Right ventricular outflow tract obstruction; DORV, Levo-double outlet right ventricle; CCTGA, Corrected transposition of the great arteries; L-TGA, Left-transposition of the great arteries; D-TGA, Dextro-transposition of the great arteries; uncertain, Uncertain survival status or uncertain clinical significance.

Based on a retrospective analysis of GDF1 function and coronary artery embryogenesis, the following hypothesis is proposed: The novel GDF1 heterozygous mutation identified in this case may partially disrupt protein function, specifically interfering with early critical steps of coronary artery development. Coronary embryogenesis begins at gestational days 32–35 in humans, with proepicardial cells and sinus venosus endothelial cells respectively differentiating into the first and second coronary vascular progenitors (1st and 2nd CVPs) (19, 20). These progenitors undergo epithelial-mesenchymal transition (EMT) to form mesenchymal cells that invade the myocardium and differentiate into the coronary system (21). By embryonic days 37–47, capillary progenitors from the proepicardial villi migrate to the aortic root to establish coronary ostia and main trunk formation, at the same time, vascular plexus-aortic anastomosis establishes arterial perfusion. During this process, GDF1 binding to TGF-β receptors exerts dual regulatory roles: enhances Smad signaling recruitment for Notch pathway activating, which guides CVP directional migration (22–24). It also directly drives EMT to generate mesenchymal cells, which forms the cellular foundation of coronary morphogenesis. Animal studies confirm that TGF-β signaling defects (TGF-βR3 knockout) lead to embryonic coronary vessel depletion and lethal developmental arrest (25, 26). Furthermore, GDF1 coordinates with angiogenic factors (VEGF, angiopoietin) to precisely regulate endothelial proliferation, lumen formation, and vascular branching architecture (27). We hypothesize that the heterozygous mutation may disrupt coronary trunk development (manifested as RCA hypoplasia) and impairs CVP migration to the cardiac surface, thereby causing microvascular remodeling (manifested as myocardial bridging). This hypothesis redefines GDF1's molecular role, extending its function beyond traditional large-vessel axial patterning to coronary-specific developmental pathways, thereby elucidating novel mechanisms underlying the pathogenesis of congenital coronary artery anomalies.

Whereas, as a single case report, a definitive causal relationship between the GDF1 variant and the phenotype cannot be established; further familial segregation analysis or functional studies would be valuable. Second, the long-term prognosis of patients with this specific genotype-phenotype correlation requires follow-up.

## Conclusion

This case reports a novel GDF1 gene frameshift mutation whose pathogenicity is supported by ACMG guidelines (PVS1 + PM2) and functional predictions (MSC95< CADD). This case represents the first documentation of GDF1 mutation associated with HCAD, expands the phenotypic spectrum of GDF1-related disorders, and provides hypothesis-generating evidence for the potential genetic mechanisms.

### Strengths and limitations

This case report has several strengths. First, it provides the first documented potential association between a GDF1 mutation and HCAD with myocardial bridging, expanding the known phenotypic spectrum of GDF1-related disorders. Second, the integration of high-quality CCTA imaging with WES and Sanger validation offers a comprehensive diagnostic approach. Third, the proposed mechanistic hypothesis linking GDF1 to coronary embryogenesis through TGF-β/Notch signaling provides a biologically plausible framework for future investigation.

However, several limitations should be acknowledged. As a single case report, this study cannot establish causality; the observed association may be coincidental, and larger cohort studies or functional validation are needed to confirm the role of GDF1 in coronary development. Second, familial segregation analysis was not performed due to the absence of available family members for genetic testing, limiting assessment of variant inheritance patterns. Third, the patient declined invasive coronary angiography, which limited the ability to obtain definitive evidence of coronary anatomy beyond CCTA or to perform functional ischemia testing, which limited the ability to obtain definitive evidence of myocardial ischemia or to exclude other potential etiologies. Fourth, functional studies (e.g., *in vitro* or animal models) were not performed to directly assess the impact of this specific GDF1 variant on coronary development. Finally, long-term follow-up data are currently limited, and the prognostic implications of this genotype-phenotype association remain unknown.

## Patient perspective

The authors confirm that written consent has been obtained from the patient for the submission and publication of this case report, including images and associated text, in accordance with the COPE guidelines.

## Data Availability

The original contributions presented in this study are included in the article/supplementary material. Further enquiries can be directed to the corresponding author/s.
